# Pharyngeal diameter in various head and neck positions during exercise in sport horses

**DOI:** 10.1186/1746-6148-10-117

**Published:** 2014-05-23

**Authors:** Li-mei Go, Ann Kristin Barton, Bernhard Ohnesorge

**Affiliations:** 1 Clinic for Horses, University of Veterinary Medicine Hannover, Foundation, Bünteweg 9, Hannover D-30559, Germany; 2 Department of Veterinary Medicine of Freie Universität Berlin, Equine Clinic, Oertzenweg 19 b, Berlin D-14163, Germany

**Keywords:** Horse, Equine pharynx, Head and neck position, Pharyngeal diameter, Upper respiratory tract

## Abstract

**Background:**

In equine athletes, dynamic stenotic disorders of the upper airways are often the cause for abnormal respiratory noises and/or poor performance. There are hypotheses, that head and neck flexion may influence the morphology and function of the upper airway and thus could even induce or deteriorate disorders of the upper respiratory tract. Especially the pharynx, without osseous or cartilaginous support is prone to changes in pressure and airflow during exercise. The objective of this study was to develop a method for measuring the pharyngeal diameter in horses during exercise, in order to analyse whether a change of head-neck position may have an impact on the pharyngeal diameter.

**Results:**

Under the assumption that the width of the epiglottis remains constant in healthy horses, the newly developed method for calculating the pharyngeal diameter in horses during exercise is unsusceptible against changes of the viewing-angle and distance between the endoscope and the structures, which are to be assessed. The quotient of the width of the epiglottis and the perpendicular from a fixed point on the dorsal pharynx to the epiglottis could be used to determine the pharyngeal diameter. The percentage change of this quotient (pharynx-epiglottis-ratio; PE-ratio) in the unrestrained head-neck position against the reference position was significantly larger than that of any other combination of the head-neck positions investigated. A relation between the percentage change in PE-ratio and the degree of head and neck flexion could not be confirmed.

**Conclusions:**

It could be shown, that the pharyngeal diameter is reduced through the contact position implemented by the rider in comparison to the unrestrained head and neck position. An alteration of the pharyngeal diameter depending on the degree of head and neck flexion (represented by ground and withers angle) could not be confirmed.

## Background

Dynamic stenosis of the upper airways occurs when the soft tissue of the upper airways can no longer withstand the high changes in pressure during exercise and structures of the nasopharynx or the larynx collapse into the lumen, thus leading to airflow obstruction [1,2]. Due to its musculomembraneous structure without osseous or cartilaginous support, the pharynx in particular is prone to changes in pressure and airflow during exercise [3].

Various studies have proven that dynamic stenotic disorders of the upper airways can only be reliably diagnosed during exercise [2,4-6]. Next to endoscopy on the high-speed treadmill, overground endoscopy provides a new method for diagnosis of dynamic disorders of the upper airways. It allows for examination under real training and competition conditions [7,8]. Furthermore, for the first time, overground endoscopy allows examination under a rider and thus also examination of the influence of the rider’s aids. In the past years, the influence of different contact positions and their effects on the sport horses’ ability and willingness to perform as well as their welfare has become the centre of a broad discussion [9,10]. As shown by Cehak et al. [11,12], dorsoventral pharyngeal diameter in horses at rest is substantially affected by head-neck position.

The aim of the present study was to examine the effects of four different contact positions on pharyngeal width in horses and, with the aid of a new method of measurement, to assess whether a change in pharyngeal diameter dependent on head-neck position can be confirmed.

## Methods

All research carried out on horses were registered and permitted (reference number Az 10A 051) by the Lower Saxonian State Office for Consumer Protection and Food Safety (LAVES, Oldenburg, Germany).

### Subject group

Twenty-one riding horses were examined within the scope of this study. All animals were warmblood horses aged between 3 and 22 years and were used for leisure riding (n = 5), showjumping (n = 8) or dressage (n = 8). Data was collected from eight mares and thirteen geldings, which showed no malfunctions of the upper airways during endoscopic examination at rest as well as during exercise. Horses with orthopaedic or cardiovascular problems or endoscopically identifiable disorders of the upper or lower respiratory tract were excluded from the study. Due to a varying level of schooling, some of the horses were used to being worked in the different contact positions while others were worked in this way for the first time during the examination.

### Endoscopic examination during exercise

For the endoscopic examination under a rider, after an individual warm-up phase, all horses were exercised according to a defined protocol. This included a short period to get used to the dynamic respiratory endoscope and the associated equipment (Dynamic Respiratory Scope, Optomed, Les Ulis, France) as well as exercise at trot and canter in four different contact positions. Per gait and contact position, two long sides of the indoor school (40 meters in length) were digitally recorded for further evaluation. As far as allowed by the horses’ level of schooling, the horses were examined in a reference position, elevation, hyperflexion and unrestrained. The investigated contact positions were mainly based on FEI guidelines. The reference position was based on the stretching posture described by the FEI, in which a low neck with the nose-line slightly in front of the vertical and the mouth reaching more or less the horizontal line corresponding with the point of the shoulder should be achieved [13]. Therefore, we asked the riders to present the horses as they would do during warming up and to minimize their influence on the posture the horse is offering and this posture was named reference position. However, due to their habitual way of training, the majority of the horses showed a posture with the nose-line slightly behind the vertical. During elevation, the poll was to be the highest point and the horse’s nose-line was to be slightly in front of the vertical. In hyperflexion, the horses were ridden as overbent and low as possible without resistance. The condition of hyperflexion was considered fulfilled as soon as the nose-line of the horse was behind the vertical. This definition is consistent with the definition by the FEI [14], even if a further subdivision of this posture has been established by now [15]. For the unrestrained head position, the riders were to loosen the reins until there was no more contact between the rider’s hand and the horse’s mouth.

All horses were ridden by one of the professional riders (A or B) belonging to the university or if desired, by the owner.

Simultaneously to endoscopy, a lateral video of the horse and rider was taken using a digital video camera (Sony DCRVX 2000, Sony Corporation, Minato, Tokyo, Japan) which was positioned in the middle of the opposite long side of the indoor school (Additional files 1 and 2).

### Evaluation

**Figure 1:**
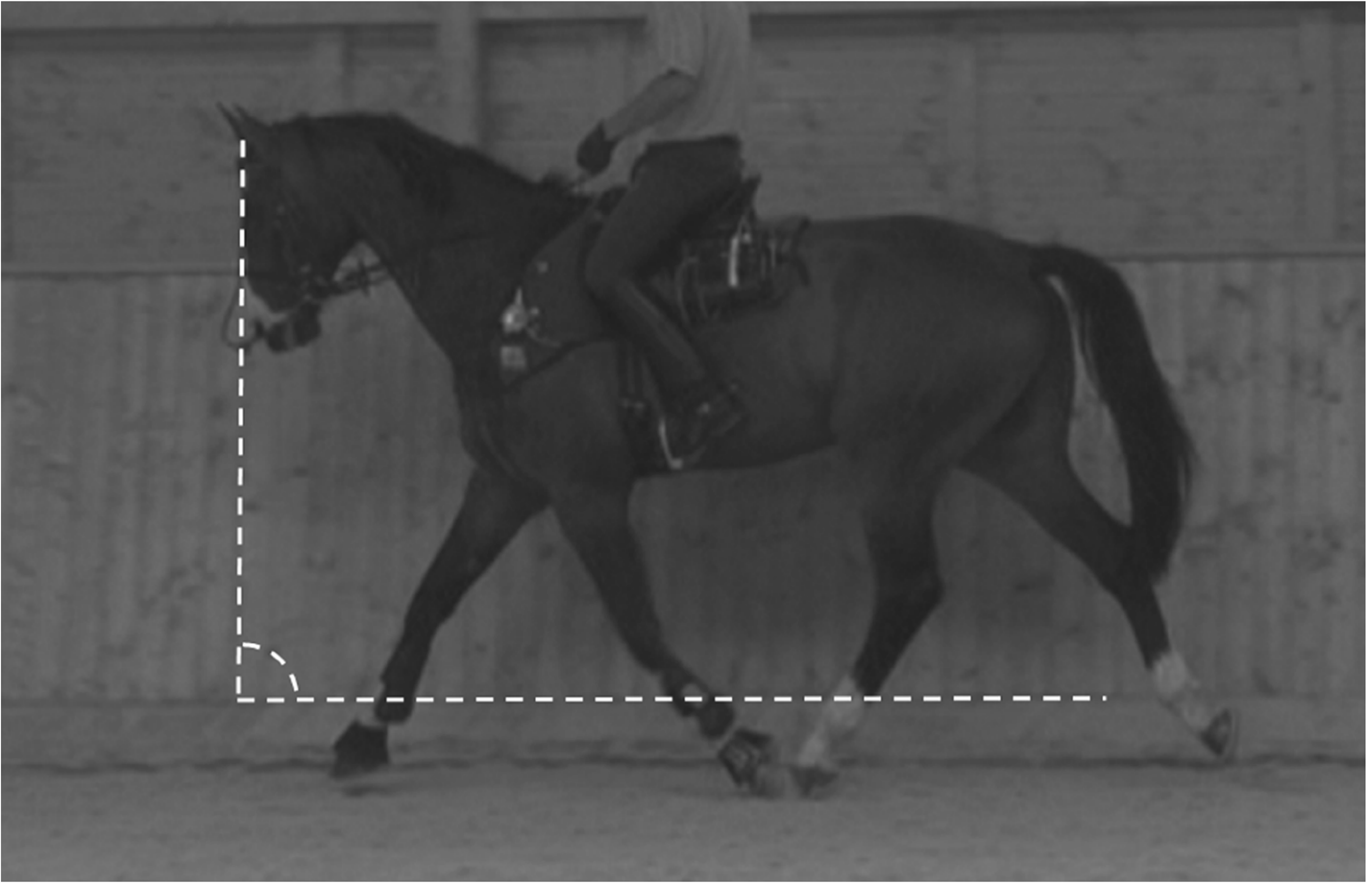
**Ground angle.** Horse during suspension phase, showing the GA defined as the angle between the ridge of the nose and a horizontal plane in the image.

**Figure 2:**
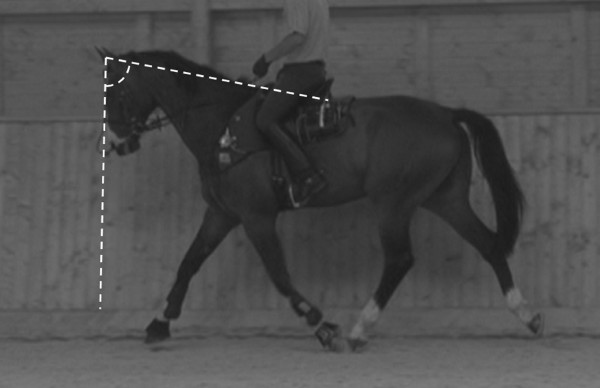
**Withers angle.** Horse during suspension phase, showing the WA defined as the angle between the ridge of the nose and the line connecting the neck and the withers.

As there were differences in contact during exercise endoscopy for each pair of horse and rider, the contact position was objectively classified afterwards based on the video footage. For this purpose, six freeze frames per long side were obtained from lateral video footage of horse and rider using a video-editing program (EDIUS 5.5, Grass Valley, San Francisco, USA). In order to be as orthograde as possible for assessment, they were taken from the video sequence while the horses were within approximately six meters of the middle of the long side. Half of the freeze frames each were taken during suspension phase or support phase respectively. For the support phase, the moment when the outside foreleg of the horse was vertical to the ground was chosen. For the suspension phase, freeze frames were taken at the moment of maximum suspension when all four feet were off the ground. The freeze frames, which were thus obtained, were used to classify objectively contact position. This was done using the program ImageJ (ImageJ 1.45e, rsweb.nih.gov/ij/) to measure the ground and the withers angle (GA and WA) in the freeze frames [16] (Figures 1 and 2).

**Figure 3:**
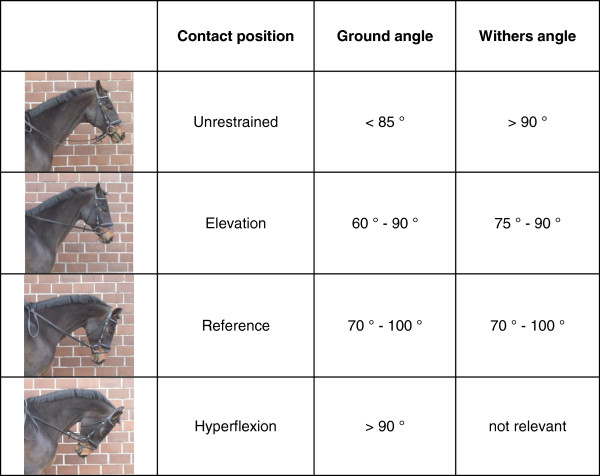
**Ranges of GA and WA for each contact position.** Each contact position was defined by a combination of GA and WA.

With the aid of the two measured angles, the four contact positions (reference, elevation, hyperflexion and unrestrained) were reviewed by defining each contact position as a combination of GA and WA (Figure 3). If the mean value of the measured angles for a horse was not within the defined range, the data of this horse for this contact position was not further analysed. Data from horses, which showed inconsistency of head-neck position during the examination of a contact position, i.e. the standard deviation of the measured angles was greater than five degrees, were also excluded.

**Figure 4:**
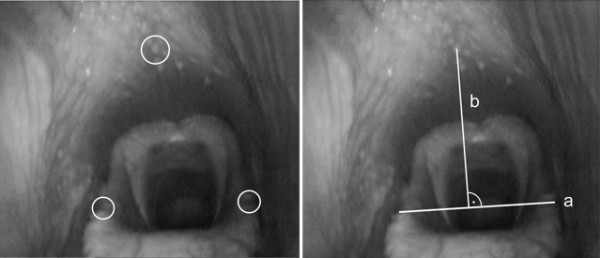
**Endoscopic images of the nasopharynx with marked fixed points.** Easily recognizable vascular patterns or follicles were used as fixed points on the dorsal pharyngeal roof and on the epiglottis (left picture). The length on the epiglottis **(a)** and the distance of the perpendicular from the fixed point on the dorsal pharynx to the reference length on the epiglottis **(b)**, are shown in the right picture. Both distances were measured and their quotient was calculated and termed pharynx-epiglottis-ratio (PE-ratio = b / a).

For the evaluation of endoscopy during ridden exercise, freeze frames from the exercise endoscopy were taken and evaluated using a video-editing program (EDIUS 5.5, Grass Valley, San Francisco, USA). These freeze frames were taken at the beginning of expiration phase, whereas breaths immediately before and after deglutition were not included. On the freeze frames of each horse, a defined length on the epiglottis as well as a point on the dorsal pharynx was determined. Easily recognizable vascular patterns or follicles were used as fixed points on all freeze frames of each horse (Figure 4). Using the graphics software program (ImageJ 1.45e, rsweb.nih.gov/ij/), the length on the epiglottis (a) as well as the distance of the perpendicular from the fixed point on the dorsal pharynx to the reference length on the epiglottis (b) were measured on the freeze frames (Figure 4). The reference length on the epiglottis (a) was chosen based on the hypothesis that in healthy horses, the width of the epiglottis remains the same during exercise and change of head-neck positions. Thus, the epiglottal width (a) served as a constant value during exercise endoscopy of the horse. In order to account for the imaging error of the measured distance (b) from the fixed point on the dorsal pharynx to epiglottal width, distance (b) between the fixed point on the dorsal pharynx to epiglottal width was divided by the respective epiglottal width (a). The quotient thus obtained was termed pharynx-epiglottis-ratio (PE-ratio), and the change in this value for the different head-neck positions was evaluated. In order to investigate the effect of respiratory phase, in ten horses, PE-ratio was calculated both during inspiration and expiration in canter in the head-neck positions examined. For comparability of the obtained PE-ratios of the different horses, relative change in mean values for the different head-neck positions was calculated. To achieve this purpose, a horse’s mean PE-ratio value for each position was divided by the PE-ratio of the respective horse in the reference position at trot. Consequently the mean PE-ratio value at trot in the reference position for each horse was equivalent to the value of 1, i.e. 100%, and thus the relative change in PE-ratio for the remaining head-neck positions could be calculated in percent. The comprehensive calculations were carried out using Access 2010 and Excel 2010 (Microsoft Corporation, Redmond, WA, USA).

Statistical evaluation of the data obtained was performed using the statistic software program SPSS Version 20 (IBM Corporation, Armonk, NY, US). The normal distribution of the obtained percentage change of PE-ratio as well as of the GA and WA was visually confirmed with the aid of Q-Q-plots and the Kolmogorov-Smirnov test. The Pearson correlation coefficient was used to check for possible paired correlations between the percentage change in PE-ratio and WA as well as GA. The effects of head-neck position and respiratory phase on the PE-ratio were analysed using a single-factor analysis of variance (ANOVA) and compared in pairs using a Tukey’s post-hoc analysis. Significance was set at p ≤ 0.01. For the ANOVA’s the power of the tests (1-β) were calculated by G*Power 3.1 [17].

## Results

In two of the horses examined, the measured GA and/or WA in all head positions analysed varied above the predetermined limit of standard deviation. They were thus excluded from further evaluation. Overall, the contact implemented by the riders only coincided with the predetermined head-neck position in 55% of the cases. The highest coincidence occurred during hyperflexion (85% at trot, 79% in canter) and at 35%, the lowest coincidence was detected at trot with elevated head-neck position. In the head-neck positions shown, the measured WA varied between 68.8° and 111° (80.3 ± 9°; mean value ± standard deviation) and the GA ranged between 57.2° and 120.2° (92.9 ± 14.2°). The smallest established value in the relative change of the PE-ratio was 0.75 and was found in the hyperflexion head-neck position at trot, whereas the largest established value was 1.75 and occurred in the reference head-neck position in canter.

Eighteen out of the 21 horses were ridden by a professional rider belonging to the university (9 by rider A, 9 by rider B) and three horses were ridden by their owners. Both professional riders were experienced and successful at the national advanced level (rider A specialised on show jumping, male, 1.83 m and 82 kg; rider B specialised on dressage, female, 1.56 m and 54 kg). The 18 horses were allocated randomly and no differences in the implementation of contact position could be detected between rider A and rider B. Both, the group of dressage horses (n = 8) and the group of show jumpers (n = 8), contained horses from the lower competition level as well as horses which were successful in competitions up to the highest level. Within the different groups of working discipline (leisure riding, showjumping and dressage) the various subgroups of age and level of schooling were too small for statistical evaluation. However, comparing the different groups of working disciplines, dressage horses showed a significant higher rate of correctly implemented contact positions.

**Figure 5:**
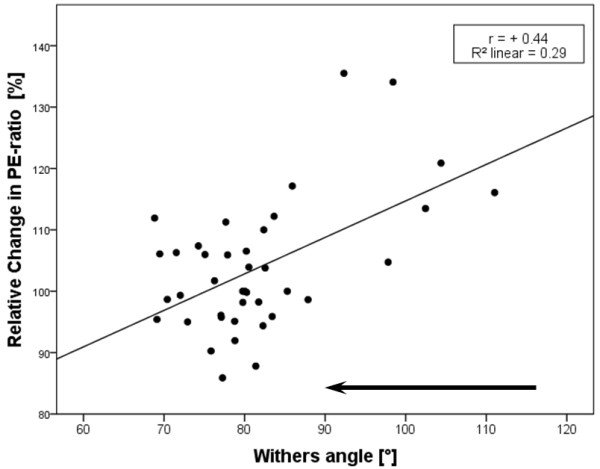
**Correlation between the WA and the relative change in PE-ratio.** The arrow (above the x-axis) points in the direction of increasing head-neck flexion (n = 40).

**Figure 6:**
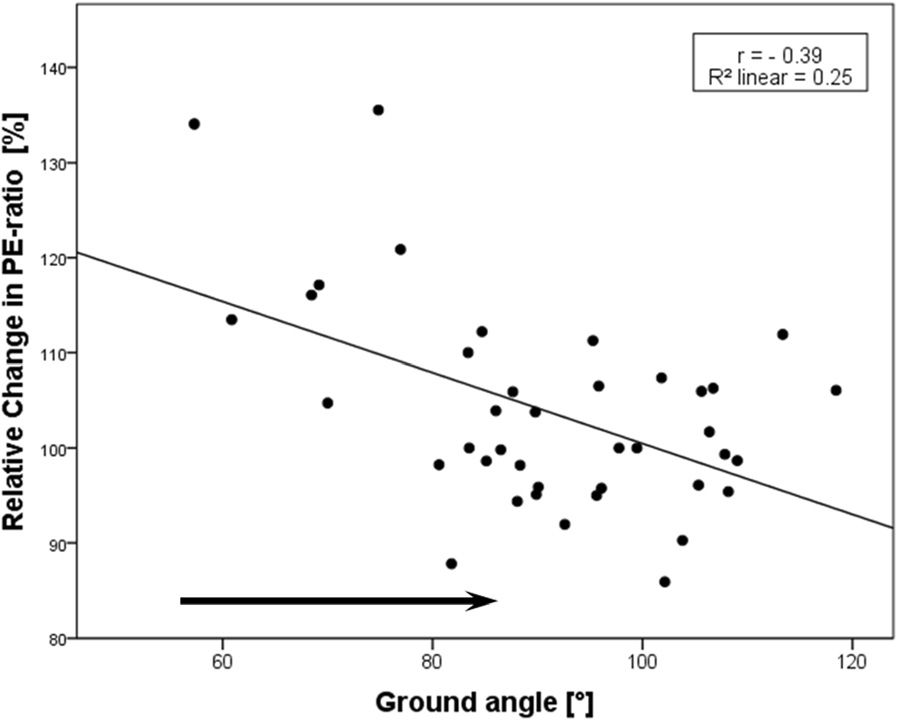
**Correlation between the GA and the relative change in PE-ratio.** The arrow (above the x-axis) points in the direction of increasing head-neck flexion (n = 40).

Only a weak, but nevertheless significant (p < 0.01), correlation between the measured angles (WA and GA) and the relative change in the PE-ratio was detected (Pearson correlation coefficient +0.44 for WA and −0.39 for GA) (Figures 5 and 6).

**Figure 7:**
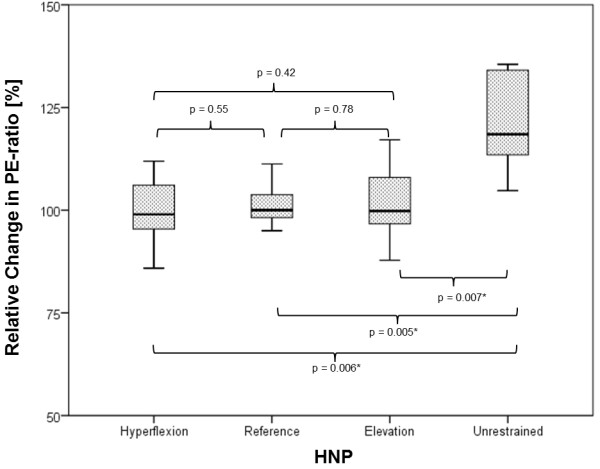
**Percentage change in PE-ratio as a function of the head and neck positions (hyperflexion, reference, elevation and unrestrained).** There was a significant difference (p < 0.01) between the relative change in PE-ratio in the unrestrained head-neck position and the other contact positions investigated. The boxes defined the upper and lower quartiles with the medians marked by the horizontal lines, the whiskers indicated the minimum and maximum values (n = 40).

A single-factor ANOVA showed that there was no significant difference between the mean values of the relative PE-ratio percentage change in the reference, elevation and hyperflexion head-neck positions in clinical unaffected horses (Figure 7). The mean value of the relative PE-ratio percentage change in the unrestrained head-neck position, however, was significantly different (p < 0.01) compared with the other examined contact positions. The subsequent power analysis yielded a power (1-β) of 95%.

**Figure 8:**
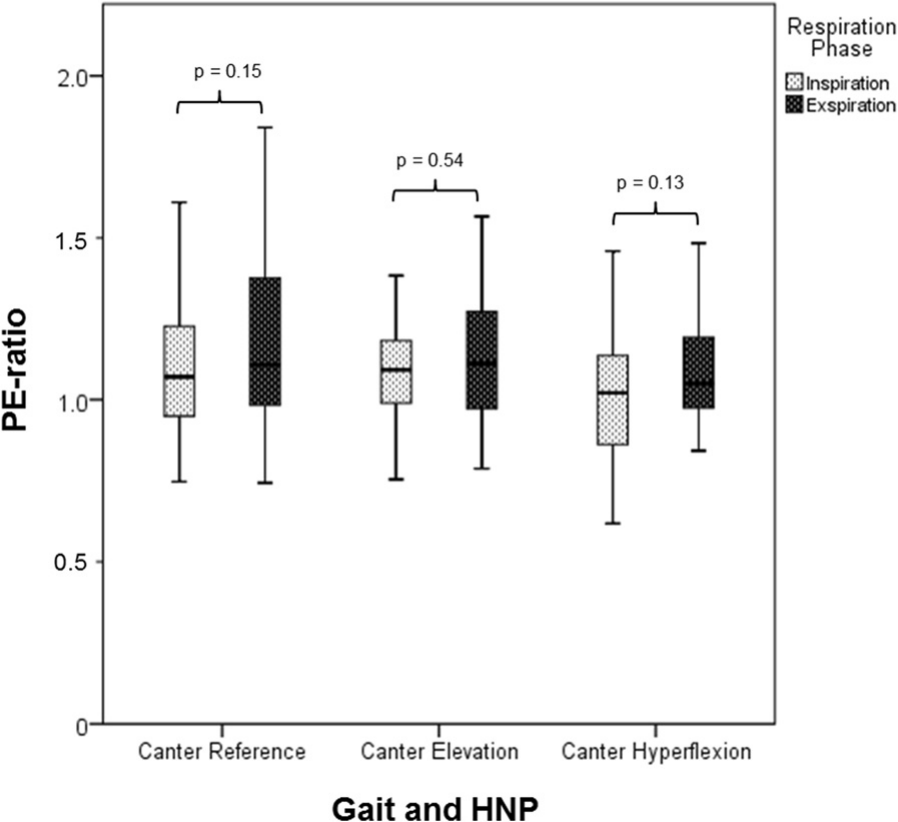
**Pharynx-epiglottis-ratio as a function of the breathing cycle.** There was no significant difference in the PE-ratio dependent on the respiratory phase in any of the examined head and neck position at canter (n = 10).

In ten horses, PE-ratio was determined during both inspiration and expiration in canter in the reference, elevation and hyperflexion positions. A single-factor ANOVA showed that dependent on respiratory phase, the mean values of PE-ratio were not significantly different in any of the examined head-neck positions (Figure 8).

## Discussion

As previously found by several authors [18-20], all horses showed very good acceptance of the endoscopic examination during exercise. Dynamic stenotic disorders of the upper airways can only be reliably diagnosed during exercise (on the treadmill or using exercise endoscopy). The influence of head-neck position on upper airway function has already been examined by many authors [21-24]. A higher incidence of dynamic stenosis could be proven particularly in a severely overbent head-neck position. A possible cause for this effect might be that, if the head is bent, the airflow no longer follows a straight line but has to make its way at an increasingly smaller angle with increasing bend [25]. Additionally there is a change in pharyngeal diameter [11,12]. To show these changes on the endoscopic image during exercise poses new challenges for evaluation. During exercise, particularly during a change of head-neck position, a rostral or caudal movement of the endoscope cannot be avoided. Due to the change in head-neck position, the soft tissue boundaries of the nasopharynx rotate around the larynx, which acts as an extension of the trachea [10]. As a consequence, there is a change in the distance of the endoscope from the larynx as well as in the angle at which the assessed structures are viewed. In addition, none of the structures viewed has a known size, and the distance of the endoscope from these structures is unknown. Thus, an absolute measurement to determine pharyngeal width is impossible, which is why the above method was developed for the present study. Under the assumption that the width of the epiglottis remains constant, this method permits to compensate for changing distances and viewing-angle between the scope and the larynx.

In the present study, only horses without any identifiable disorders of the upper respiratory tract were included, in order to test the new measuring method. In ten horses, the PE-ratio in the different head-neck positions in canter was determined during both expiration and inspiration. These ten horses were selected, because of the high quality of their endoscopic videos, in which the fixed points were easily visible during the entire breathing cycle. No significant difference with regard to the respiratory phase could be detected. Opposed to this, in horses with dynamic pharyngeal collapse the pharyngeal walls will collapse during inhalation [26] and a detectable difference in the PE-ratio measured during inspiration and expiration could be expected. Due to the higher quality of the images, for further evaluation freeze frames from the endoscopic videos were exclusively taken during expiration in all horses.

Through the simultaneous start of recording exercise endoscopy as well as lateral video footage, it was ascertained that the freeze frames, which were to be assessed were obtained within the respective contact position to be investigated. A completely synchronous extraction of freeze frames from exercise endoscopy and lateral video footage was not possible since not all analysable freeze frames from exercise endoscopy corresponded with orthograde lateral images of horse and rider. Measurement of the GA and WA enabled objective assessment and verification of the different head-neck positions, even if different riders presented the horses and the required contact positions were implemented differently.

The selected contact positions were derived from the FEI guidelines [13]. For the purposes of the present study, the head-neck positions were selected to be practicable for most of the horses, even if they are previously not trained accordingly. Thus, the used contact positions had to be slightly modified and we laid down a new nomenclature. Figure 3 shows the reviewed contact positions and their accompanying combination of GA and WA. Note that in the case of hyperflexion only the GA is decisive for definition. The FEI states that hyperflexion is a working technique to provide a degree of longitudinal flexion of the neck [14]. Therefore, the condition of hyperflexion could be considered fulfilled if the nose-line of the horse is behind vertical (GA > 90°). In contrast, the extreme yielding of the poll in the sense of the so-called “Rollkur”, as described by Meyer et al. [9], is only achieved if the nose-line is 20 degrees and more behind the vertical. In the present study, only two horses could show this posture.

A significant difference in the relative change in PE-ratio between the unrestrained head position and the other head-neck positions investigated could be confirmed. Similar results were already reported [21-24,27]. In all these studies, the horses were examined in an unrestrained head position and with side-reins or in a contact position determined by the rider respectively. The aim of our study was to differentiate further these head-neck positions previously only described as contact, and to investigate their effect on pharyngeal width. Contrary to the hypothesis that PE-ratio would be smallest in hyperflexion, a change in the percentage PE-ratio corresponding to the increasing degree of contact could not be confirmed. A possible explanation might be the choice of horses and their largely varied level of schooling. Thus, some of the horses were ridden in elevation and/or hyperflexion for the first time in the course of this study.

In addition, the fact, that only approximately half of the data could be further evaluated after assessment of the head-neck position is due to a relatively heterogeneous subject group in terms of age and level of schooling. Half of the freeze frames from the lateral video footage of horse and rider each were obtained in suspension phase or support phase respectively. In the elevation head-neck position, particularly young horses tended to lean on the bit during the support phase due to their insufficient self-carriage, thus getting their nose-line markedly further behind the vertical than during suspension phase. Hence, the variation in GA and WA was above average and for at least one of those angles the predetermined limit of the standard deviation was exceeded. Within each group of working discipline, the groups of varying age and level of schooling were too small for a well-founded statistical statement. However, the group of dressage horses showed a significant higher rate of correctly implemented contact positions compared to the other two groups. This may be an indication of the impact of the level of schooling. No significant dependence between the choice of rider (A or B) and the proper performance of contact positions could be detected.

In the present study, the choice of the ranges of angles used to define the different head-neck positions (Figure 3) still needs to be critically assessed. Particularly, in the reference head position the chosen ranges of angles overlap with those of hyperflexion and elevation. For performing the reference head position the riders were simply asked to ride the horses similar to a common warm-up-phase. Ideally, an extension posture with a low neck and the nose-line slightly in front of the vertical should have been achieved. However, the majority of the horses were presented with the nose-line slightly behind the vertical, which leads to an overlap with the definition of hyperflexion. This probably stemmed from the way they were used to be ridden. The change in PE-ratio was investigated in relation to the reference position in order to enable a comparison between the horses. Hence, the outcome of the present study may be influenced by the choice of investigated contact positions and we cannot completely exclude, that a choice of head-neck positions defined by clearly distinguished ranges of angle may have led to a deviating result. Furthermore, the results of our study are based on the assumption, that the width of the epiglottis remains unaltered regardless of changing head-neck positions. A further verification of this hypothesis was not possible in the course of our study.

Between the calculated percentage change in PE-ratio and the measured angles (GA and WA) only a weak correlation could be confirmed. A possible explanation might be, that in this study, head-neck positions were merely defined by two angles in the sagittal plane and the level at which the horse’s head was held at that time was not taken into account. Thus, the degree of flexion was converted to a measurable unit, but the level of head and neck in space described as “Einstellung” by Meyer et al. [9] was not recorded. However, Cehak et al. [11] showed in their study that in horses at rest, there is a higher correlation between pharyngeal width and the degree of flexion than between the pharyngeal width and the level of head and neck in space. Likewise, a previous study showed that in horses at rest, there is a significant correlation between the radiographic pharyngeal diameters and the GA or WA applied respectively [16].

Furthermore, it is apparent that in the study on horses at rest, the determined pharyngeal diameter in the flexed head-neck position was significantly smaller than in the other two head-neck positions examined (neutral and extended). In the present study on horses during exercise, on the other hand, a significant difference between the unrestrained head-neck position and the other head-neck positions examined was proven, but not between the different degrees of contact. An explanation for this is the changing muscular tone of the upper airways during exercise. As there is no osseous or cartilaginous basic support structure particularly in the caudal part of the nasopharynx of the horse, its stability is solely dependent on the tone of the associated muscles. The stylopharyngeal muscle, upon contraction, elevates the dorsal pharynx and prevents a pharyngeal collapse [28]. Its activity is modified by the glossopharyngeal nerve [28]. Upon increased respiration during exercise, mechanoreceptors in the upper airway mucosa are activated and lead to a higher activity of the muscles of the upper airways [29]. Due to the general assumption that sedation leads to a change in muscular function and because the mechanoreceptors are not activated at rest, the results of this study, where horses were examined during exercise, differed from those of previous studies, where sedated horses were examined at rest.

## Conclusions

In conclusion, we succeeded in providing an initial approach to evaluate pharyngeal opening objectively. Under the assumption that the width of the epiglottis remains constant, the present study provides a method to assess dimensions of the upper airway independently of the viewing-angle and distance between the scope and the relevant structures. The percentage change in the PE-ratio permitted us to perform an analysis between different horses. A pharyngeal constriction due to the contact implemented by the rider in comparison to the unrestrained head-neck position could be confirmed. This corresponds with the results of other studies comparing the unrestrained head-neck position with a chosen contact position. A further constriction dependent on the degree of flexion, however, could not be proven in clinically healthy horses. Thus, the hypothesis that increasing hyperflexion leads to an increasing constriction of the pharynx, could not be confirmed. Due to the available subject group, the question whether extreme overbending, as it is presently discussed in the riding community, leads to a further decrease in pharyngeal width could not be answered conclusively. In addition, it will be subject of further studies to examine the effect of rider induced contact positions on the change in the PE-ratio in clinically affected horses suffering from dynamic pharyngeal collapse.

## Abbreviations

GA: Ground angle; WA: Withers angle; PE-ratio: Pharynx-epiglottis-ratio.

## Competing interests

None of the authors has any financial or personal relationships, which could inappropriately influence or bias the content of this paper.

## Authors’ contributions

LG designed the study, carried out the examinations, analysed data, drafted and wrote the manuscript. AKB contributed to the study design and helped to draft the manuscript. BO contributed to the study design, data analysis and interpretation. All authors read and approved the final manuscript.

## Supplementary Material

Additional file 1 **Edited video at trot.** Endoscopic video and synchronous lateral video of horse and rider at trot during one long side of the indoor school. Click here for file

Additional file 2 **Edited video at canter.** Endoscopic video and synchronous lateral video of horse and rider at canter during one long side of the indoor school. Click here for file
